# Unveiling molecular mechanisms and candidate genes for goss’s bacterial wilt and leaf blight resistance in corn through RNA-Seq analysis

**DOI:** 10.1186/s12864-025-11830-4

**Published:** 2025-08-18

**Authors:** Mohammad Sayari, Sara Victoria Good, Dimitri Trubetskoy, Mohamed El-Shetehy, Aria Dolatabadian, Atta Soliman, Arash Kheirodin, Fouad Daayf

**Affiliations:** 1 Department of Plant Science, University of Manitoba, Winnipeg, R3T 2N2 MB Canada; 2 Department of Biology, The University of Winnipeg, 515 Portage Avenue, Winnipeg, MB R3B 2E9 Canada; 3 Department of Biological Sciences, University of Manitoba, 50 Sifton Road, Winnipeg, MB R3T 2N2 Canada; 4 Department of Botany and Microbiology, Faculty of Science, Tanta University, Tanta, 31527 Egypt; 5 School of Biological Sciences, The University of Western Australia, Crawley, WA Australia; 6 Department of Entomology, Texas A&M AgriLife Research and Extension Center, Dallas, TX 75252 USA; 7 Department of Genetics, Faculty of Agriculture, University of Tanta, Tanta, 31527 Egypt

**Keywords:** Goss's bacterial wilt, Leaf blight disease, *Clavibacter nebraskensis*, Resistance, RNA-Seq, Differential gene expression

## Abstract

Goss’s bacterial wilt and leaf blight (GWLB) induced by *Clavibacter nebraskensis* poses a significant threat to corn production in Canada and the US. We conducted RNA-Seq analyses on two corn hybrid lines with contrasting levels of susceptibility to GWLB, 447 (susceptible) and 450 (resistant), to elucidate the molecular mechanisms underlying corn resistance to the disease. The two selected corn lines were subjected to inoculation with two isolates of *C. nebraskensis* exhibiting distinct levels of aggressiveness: DOAB232 (DOAB), a weakly aggressive isolate and Cn14-5-1 (BACT), a highly aggressive one, or were left untreated (CTL) Total RNA was isolated from leaf tissue in three biological replicates for each hybrid x treatment combination five days post-inoculation (dpi), a timepoint selected to capture the early transcriptional response to pathogen infection. RNA-sequencing was performed following poly-A tail isolation of the RNA on all 18 samples (6 combinations * 3 replicates). Following data cleaning and filtering of the raw RNA-seq data, unsupervised clustering, differential expression and pathway analysis was conducted. Our analysis revealed extensive transcriptional reprogramming in both resistant and susceptible lines, particularly in the susceptible corn line (447) when inoculated with the either the weak or aggressive strain of *C. nebraskensis*, but also in the resistant corn line when inoculated with the aggressive bacterial isolate. Inoculation of the resistant corn line (450) to the aggressive *C. nebraskensis* isolate, led to increased expression of photosynthesis-related and defense-associated genes, and downregulation of secondary metabolism and stress responses pathways, while inoculation of the susceptible corn line (447) with the weakly aggressive *C. nebraskensis* isolate led to upregulation of defense-associated genes. The functions of some of the top differentially expressed genes in each comparison are discussed within the context of understanding the molecular underpinnings of corn resistance to GWLB disease to identify targets for genetic enhancement towards higher disease resistance levels.

**Supplementary Information:**

The online version contains supplementary material available at 10.1186/s12864-025-11830-4.

## Introduction

Corn (*Zea mays* L.) is one of the most important crops globally, and it provides food, animal feed, and raw materials for various industries [1]. However, the production of corn faces many challenges due to both biotic and environmental factors. One such challenge is Goss’s bacterial wilt disease (GWLB) caused by *Clavibacter nebraskensis*, a Gram-positive bacterium [2]. This disease threatens corn cultivation worldwide, and causes substantial yield losses, particularly in susceptible corn hybrids [3]. The increasing prevalence of GWLB, compounded by warmer temperatures, as a disease in North America underscores the need for accessible and improved methods of disease control [4]. Efforts are ongoing to explore environmentally friendly approaches for disease management. Currently, there are no chemical treatments for GLWB and the most commonly used strategies to reduce crop loses revolve around cultural practices like crop rotation and planting of resistant varieties [5]. However, it is important to acknowledge that these strategies have limitations and are not always entirely effective in controlling the spread of the disease.

Given the limitations of current management practices, recent research has increasingly focused on understanding the genetic and molecular basis of resistance to GWLB in corn [6]. Efforts to improve resistance breeding have leveraged molecular tools such as gene expression profiling, identification of resistance-associated markers, and functional genomics approaches. These environmentally friendly strategies aim to enhance our understanding of host-pathogen interactions and enable the development of durable resistance in elite corn lines. Several molecular approaches have been proposed for managing Clavibacter-related bacterial wilt disease. These include the use RNA interference (RNAi) technology to suppress the expression of corn genes inoculated with *C. michiganensis subsp. nebraskensis* [7], the development of molecular markers for breeding resistant corn lines [8, 9], and the application of micro-Raman spectroscopy under laboratory conditions for the rapid and accurate detection of *C. michiganensis subsp. michiganensis* [10]. Integrating such advanced detection methods into comprehensive disease management strategies holds promise for enhancing our ability to effectively detect and respond to Clavibacter infections, thereby contributing to developing more resilient corn crops. The complexity of the genetics underlying resistance to Goss’s wilt in maize is further elucidated by the study by Singh et al. [11]. Employing a genome-wide association analysis, these researchers identified key genetic factors associated with resistance to Goss’s wilt. Their findings revealed a complex genetic architecture governing maize’s response to the pathogen, highlighting the involvement of multiple loci in conferring resistance.

Several other studies have investigated the molecular basis of resistance and susceptibility in corn lines to GWLB using gene expression analysis approaches. For example, we elucidated key aspects of corn resistance to *C. nebraskensis* by investigating the involvement of signaling pathways [12]. This research showed that Goss’s wilt resistance in maize is mediated by the activation of salicylic acid (SA) and programmed cell death (PCD), highlighting the central role of these mechanisms in developing resistance. In another study, Egreja [13] conducted a transcriptome-wideanalysis of maize in response to *Xanthomonas vasicola pv: vasculorum* and *C. nebraskensis*. Although the study primarily focused on two bacterial pathogens, it uncovered distinct molecular responses to each bacterial challenge. Another research by Yokotani et al., [14] elucidated the transcriptomic landscape of *C. michiganensis subsp. michiganensis*-infected tomatoes, and identified a significant role of salicylic acid pathway in modulating the host’s defense mechanisms.

Understanding the molecular mechanisms underlying pathogen invasion, host defense, and resistance/susceptibility in corn is essential for developing effective and environment-friendly approaches for controlling GWLB. Transcriptome analysis using high-throughput RNA sequencing (RNA-Seq) has emerged as a powerful tool for investigating gene expression changes and regulatory networks in response to stress and disease in plants [15]. While other studies have used RNA-sequencing to investigate gene expression changes in corn in response to GWLB infection [6, 12], none have compared gene expression profiles between GWLB-resistant and susceptible corn hybrids inoculated with bacterial strains with contrasting levels of aggressiveness in a comprehensive way.

In this study, our objectives were (i) to use RNA-Seq to compare gene expression profiles of GWLB-resistant and susceptible corn hybrids in response to *C. nebraskensis* strains with contrasting levels of aggressiveness, and, as a result of such comparison, (ii) to identify differentially expressed genes (DEGs) associated with GWLB resistance or susceptibility, and then (iii) to conduct gene ontology (GO) enrichment analysis to gain insights into the biological processes and molecular functions associated with GWLB resistance or susceptibility in corn. The overarching goal of the study is to provide new insights into the genetic basis of GWLB resistance in corn and identify potential targets for developing new resistant hybrids.

## Materials and methods

### Plant materials

Two corn hybrid lines, 447 and 450 (kindly provided by Dr. Lana Reid, AAFC Ottawa, Canada), were used in these experiments. The 447 line is susceptible, while 450 is a resistant hybrid line to GWLB and leaf blight disease. Corn plants were grown in metro mix LA4 (Agro SunShine) in a growth room under a photoperiod of 16 h light/8 hours dark with 60% humidity for four weeks up to the V4-V5 leaf stage.

### Bacterial isolates and inoculation

Two *C. nebraskensis* isolates with contrasting aggressiveness levels, DOAB and BACT, which are weakly and highly aggressive isolates, respectively, were selected [17]. The isolates were streaked on NBY (Nutrient Broth Yeast extract) medium plates and then incubated at 26℃ for three days. Cells were harvested using phosphate buffer and the concentration was adjusted by spectrophotometry to OD_600_ = 0.5 according to Soliman et al., [16]. Corn plants were inoculated using leaf inoculation method [16], followed by disease assessments specific to each method. Leaf tissue from three resistant and three susceptible corn lines inoculated with either the highly or weakly aggressive Cn isolates (2 corn lines * 2 isolates * 3 replicates = 12 biological replicates) as well as three non-symptomatic-controls (inoculated with phosphate buffer, 2 corn lines = 6 biological replicates) were collected five days following inoculation, immediately frozen in liquid nitrogen and stored at – 80 ℃. This early timepoint provides insight into the early molecular events triggered by the pathogen, allowing us to better understand the gene expression changes that occur in both resistant and susceptible corn lines prior to any visible disease symptoms. Inoculated leaves from the same plant were pooled together for each biological replicate.

### RNA isolation and sequencing

Total RNA was isolated from the frozen tissues using the RNeasy Plant Mini Kit (QIAGEN, Inc., Valencia, CA) following the manufacturer’s instructions. Total RNA was quantified using a NanoDrop 2000 Spectrophotometer, and the quality of the RNA was assessed using agarose gel electrophoresis. High-quality RNA from 18 individuals (447BACT, 447DOAB, 447CTL, 450BACT, 450DOAB and 450CTL; three replicate each) was sent to Genome Quebec (Centre d’expertise et de services Génome Québec, Montréal (Québec) Canada) following the facility’s instructions. The RNA samples passed quality control, and following isolation of RNA harbouring poly-A tails (which includes mRNA and most long non-coding RNAs (lncRNAs), stranded libraries were constructed using the Illumina TruSeq Stranded mRNA Library Prep Kit. The libraries were then indexed, quantified, and pooled in equimolar concentrations before being sequenced on an Illumina HiSeq 4000 system with 100 bp paired-end (PE) reads.

### RNA-Seq analysis

Differential gene expression was assessed through six comparisons, as outlined in Table 1. RNA-Seq paired-end (PE) reads were checked for quality using FASTQC [17] and adapter sequences trimmed with Trimmomatic (v 0.32) [18] (parameters: ILLUMINACLIP: TruSeq3-PE-2.fa: 2:15:10 LEADING: 5 TRAILING: 5 SLIDING WINDOW: 4:5 MINLEN: 50).We used the genome assembly and associated gene annotation file available for *Zea mays* from NCBI available at: https://www.ncbi.nlm.nih.gov/assembly/GCF_902167145.1. This assembly includes ten nuclear chromosomes, the mitochondria and chloroplast genomes, as well as 675 unassembled scaffolds. A total of 34,337 protein-coding genes, which collectively encode 57.578 proteins are included in the annotation. Trimmed PE fastq files were aligned to the genome using HISAT2 (v 2.1.0) (hisat2 –p8 –dta –x index_file-1 read_pair1.fastq − 2 read_pair2.fastq–S output.sam) [19]; the resulting SAM files were indexed, sorted, and converted to BAM format using Samtools (v 1.9, http://www.htslib.org/). Mapped reads were assembled into transcripts using the StringTie (v 2.0) algorithm and the -e parameter [19]. The number of fragments per kilobase of transcript per million mapped reads (FPKM) value for each gene was calculated using cufflinks (version 2.2.1); only those genes with an FPKM value ≥ 1 in at least one sample were retained for further differential gene expression analyses. For downstream clustering analyses – including principal component (PCA) and multi-dimensional scaling (MDS) analyses, the FPKM counts were transformed using log2(CPM + c) where c was set to 4 with the cpm function in EdgeR [20], and missing values were assigned the median value for the gene.

**Table 1:** Comparisons in DEGs analysis

**No.**	**Sample1**	**Sample2**	**Comparison Description**
**1**	447BACT	447CTL^a^	Effect of highly aggressive bacteria on susceptible corn line
**2**	450BACT	450CTL	Effect of highly aggressive bacteria on resistant corn line
**3**	447DOAB	447CTL	Effect of weakly aggressive bacteria in susceptible corn line
**4**	450DOAB	450CTL	Effect of weakly aggressive bacteria in resistant corn line
**5**	450DOAB	447DOAB	Effect of weakly aggressive bacteria in resistant vs susceptible corn line
**6**	450BACT	447BACT	Effect of highly aggressive bacteria in resistant vs susceptible corn line

^a^*CTL* Untreated control plants

### Cluster analyses

To depict the global gene expression patterns across samples, we first generated a scree plot, which indicated that 86.8% of the variation in the data was explained by the first two components of a PCA analysis (Supp Fig. S1A). The top 2000 genes with the highest standard deviation across treatments were used to generate both a hierarchical and k-means clustered unsupervised heat map with k = 6 clusters based on the results of the k-means elbow plot (Supp Fig. S1B). We then opted to perform a multi-dimensional scaling (MDS) analysis to depict the relationship among samples in global gene expression patterns since it preserves the original distances between data points and does not employ dimension reduction per se.

### DEGs analysis

DEGs analysis was performed for the six contrasts listed in Table 1 using DESEQ2 [21] on the non-transformed data as DESEQ2 employs its’ own transformation algorithm. Genes with a log2 fold change > 1.0 and false discovery rate (FDR) < 0.05 were retained. DEGs was visualized with volcano plots using the R-package Enhanced Volcano. The clustering and DEGs analyses were performed in the R platform v 4.0.2.

### Pathway analysis

A pathway analysis was performed using Generally Applicable Gene-set Enrichment (GAGE) [22]. GAGE is based on a parametric gene randomization procedure. It is robust to independent attributes, including differences in sample size, assay platform and other sources of heterogeneity. GAGE pathway analysis was conducted on all genes exhibiting a consistent direction of expression across all studies with an FDR < 0.2 and was used to identify co-regulation of experimental gene sets using the direction (up or down) of fold changes.

### Gene ontology enrichment analysis

Genes that were differentially expressed at an FDR threshold of < 0.1 were selected for GO enrichment analysis. GO term enrichment was performed using the Maize Genetics and Genomics Database (MaizeGDB) via the MaizeMine platform (https://maizemine.rnet.missouri.edu/maizemine/begin.do). The gene sets were uploaded, and enrichment analysis conducted using the categories for biological processes, molecular function, and cellular component. The statistical significance of GO terms was calculated using a hypergeometric test, and the results were corrected for multiple testing using the Benjamini-Hochberg method. GO terms with a corrected *p*-value (FDR) < 0.05 were taken to be significantly enriched.

### Validation of the results using qRT-PCR analysis

The same plant tissues used for RNA sequencing were used for validation experiments. TRI Reagent (Invitrogen) was used to extract total RNA following standard protocols. The extracted RNAs were treated with DNase I (RNase free, Promega) to remove leftover DNAs. The High-Capacity Reverse Transcription Kit (Applied Biosystems) synthesized cDNA according to the manufacturer’s protocol. Real-time quantitative PCR (qRT-PCR) was conducted using a SYBR green kit (PowerUp SYBER Green Master Mix (ThermoFisher Scientific, USA) according to manufacturer protocol. The PCR thermal parameters were as follows: pre-denaturation at 94 ◦C, 30 s, one cycle; 94 ◦C, 10 s, 60^◦C^, 30 s, 35 cycles. The relative gene expression was determined using the 2^−ΔΔCT^ method [23], with both actin and beta-tubulin serving asas reference genes, and the control treatment was used as a calibrator. The global mean of both housekeeping genes was used for analysis. The genes chosen for validation were selected based on their high differential expression, functional relevance, and potential role in plant defense, stress response, or metabolic pathways associated with resistance mechanisms. Priority was given to genes with the highest fold-change values and those exhibiting consistent differential expression across the four treatment groups (447-BACT, 447-DOAB, 450-BACT, and 450-DOAB) compared to un-inoculated control plants. Additionally, some genes were randomly selected from the list of highly upregulated genes to ensure broader representation. The qRT-PCR data was analyzed using the one-way ANOVA with a post hoc least significant difference (LSD) test (*P* < 0.05) using the Statistical Analysis Software (SAS) (Version 9.1 for Windows; SAS Institute, Cary, NC, USA) to identify which pairwise comparisons were statistically significant.

## Results

### Transcriptome data overview

**Fig. 1:**
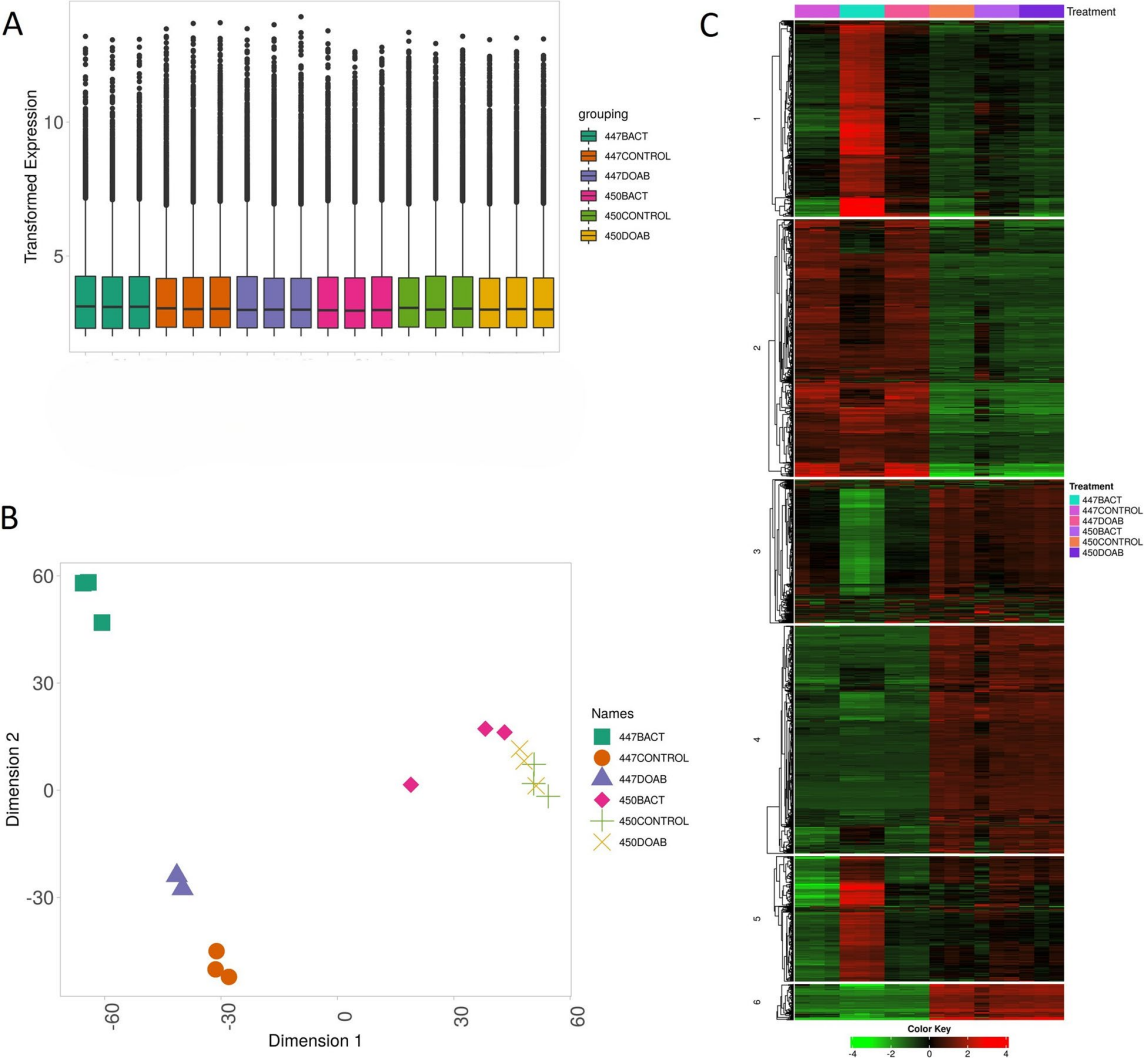
**A** Boxplot of transformed gene expression counts across all 18 RNA-seq samples. Raw FPKM values from Cufflinks were converted to counts per million (CPM) using the cpm function in EdgeR. Only genes with CPM >  1 across all samples were retained for further analysis. Each box represents a biological replicate grouped by cultivar (447 or 450) and treatment (CONTROL, DOAB, or BACT), as indicated in the color legend. **B** Multidimensional scaling (MSD) plot of the global gene expression among all transformed gene counts among all 18 samples. Samples from the resistant corn line 450 clustered closely together across treatments, whereas the susceptible line 447 showed greater separation, particularly in the BACT treatment, indicating strong transcriptional reprogramming. **C** k-means clustering plot showing the clustering of genes and treatments using k = 6, based on the k-means elbow plot. Samples from each of the six treatments are grouped, and all treatments for 447 vs. 450 corn lines are clustered together. Distinct clusters of gene expression patterns were observed amongst different samples and treatments. These clusters reflect major differences in transcriptional response between the susceptible and resistant corn lines under different bacterial challenges

Three replicates of 4-week-old inbred corn lines 447 and 450, which are susceptible and resistant to *C. nebraskensis*, respectively, were inoculated with BACT and DOAB, known as highly aggressive and weak strains of *C. nebraskensis*, respectively. Disease symptoms were observed five days after inoculation, and leaf samples were collected on the 5th day. Total RNA was extracted, quality checked and used to prepare mRNA-libraries and perform RNA-sequencing of all protein-coding transcripts. The total number of reads obtained per sample ranged from 20.5 to 24 million reads with a median value of 22.4 million reads and a similar transformed expression value (Fig. 1A). Leaf samples from corn lines 447 or 450 inoculated with either sterile water (control), or *C. nebraskensis* isolates (BACT or DOAB), and were labeled as: 447CTL, 447BACT, 447DOAB, 450CTL, 450BACT and450DOAB, respectively. Of the 34,337 protein genes annotated in the *Zea mays* genome, non-zero counts were obtained for 24,876 genes. (Supplementary File S1).

### Unsupervised clustering analyses

Unsupervised hierarchical clustering of the top 2000 genes with the highest standard deviation in expression across all 18 samples was performed using the transformed data. This analysis sorted the samples into their correct treatment and separated the expression of all samples between corn line 447 and 450 (Supp Fig. S1C). One cluster consisted of genes upregulated across all three treatments of the susceptible corn line 447 relative to the resistant corn line 450, another the opposite (upregulated in 450, downregulated in 447) and the third cluster highlighted the large number of genes that were upregulated in the 447 susceptible corn line infected with the highly aggressive bacterial strain BACT (Supp Fig.S1C). The larger transcriptional response of the susceptible corn line 447 in response to infection with the aggressive bacterial challenge (447BACT) was also apparent in the MDS plot, which showed similarity in the global gene expression among all treatments for the resistant corn line 450 but greater variation among treatments for the susceptible corn line 447 (Fig. 1B). Comparison of the global correlation in gene expression between all samples indicated that the expression of genes between all 450 treatments were correlated at > 0.96, and were > 0.99 for most samples. In contrast, the correlation in gene expression between treatments of corn line 447 was lower between those infected with DOAB vs. BACT or CTL, reflecting the induction of different gene expression pathways in the 447BACT treatment (Supp Fig.S2).

Using a k-means clustering approach, we generated a heatmap using k = 6 clusters (Fig. 1C); Inspection of the clusters showed the same three main patterns of gene expression as identified in the hierarchical analyses. Large differences in gene expression were apparent between corn lines 447 and 450, and the susceptible corn line exhibited greater changes in expression based on whether it was exposed to the mild or aggressive bacterial strain (Fig. 1C).

### DEGs associated with exposure to highly and weakly aggressive strains of ***C. nebraskensis*** in resistant (450) and susceptible (447) corn lines

**Fig. 2:**
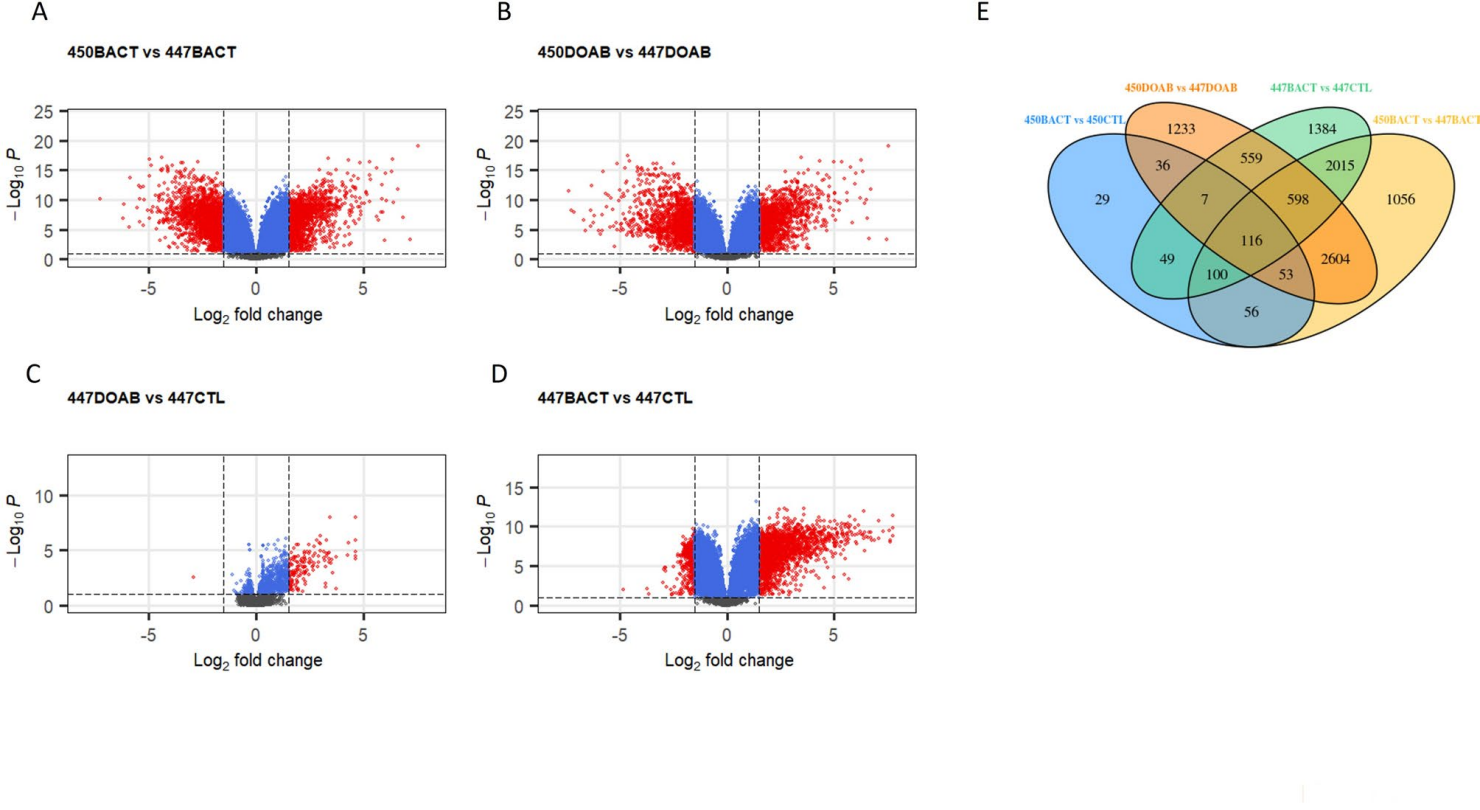
**A**-**D** Volcano plot of the DEGs between control maize and maize inoculated with aggressive (BACT) or weak (DOAB) strains of *C. nebraskensis* after five days. Up and down (red) regulated genes that had a log2fold change > 1.5 and an FDR *p*-value < 0.1 are coloured, while those that are coloured blue had an Adjusted *p*-value < 0.1, but a fold change < 1.5. E) Venn diagrams showing the number of shared differentially expressed genes between four comparisons: 450BACT vs. 450CTL (blue), 450DOAB vs. 447DOAB (orange), 447BACT vs. 447CTL (green) and 450BACT vs. 447BACT (yellow)

Using the non-transformed counts of all genes with non-zero counts, differential gene expression between 6 treatments was performed in DESEQ2 (Supplementary Table S1, data in Supplementary File 1). Overall, few DEGs were found for resistant corn line 450 infected with the weakly aggressive (5 genes for 450DOAB vs. 450CTL) strain of bacteria, and 446 genes were found to be differentially expressed in corn line 450 when inoculated with highly aggressive bacteria (450BACT vs. 450CTL, Supp Fig. S3, Table S1). However, a total of 6598 DEGs were identified in 450BACT vs. 447BACT (Fig. 2A) and 5206 differential expressed genes were identified when comparing 450DOAB vs. 447DOAB (Fig. 2B). Comparing the change in expression of the susceptible corn line infected with the same bacterial strain, 314 DEGs were identified when the susceptible corn line 447 was infected with the weakly aggressive bacteria (447DOAB vs. 447CTL, Fig. 2C) and 4828 DEGs were identified when 447 was infected with the highly aggressive bacteria (447BACT vs. 447CTL, Fig. 2D). Visualization of the DEGs on volcano plots further highlights that treatments exhibiting the greatest difference in gene expression were the response of the susceptible 447 corn line, to the highly aggressive bacteria (BACT) as well as the overall response of the susceptible 447 corn line to any infection relative to the 450 resistant corn line, which exhibited little change in gene expression across treatments (Fig. 2A-D, Supp Fig. S3).

Next, we identified the number of differentially expressed (up or down-regulated) genes in common between treatments with the aggressive (BACT) or mild (DOAB) bacterial strains (Fig. 2E) using a Venn diagram that focused on the four treatments with the most DEGs (all except 450DOAB vs. 450CTL and 447DOAB vs. 447CTL) Overall, 116 genes were differentially regulated across all four treatments. 2604 genes were differentially regulated in the susceptible (447) vs. resistant (450) corn lines in response to either the mild (DOAB) or aggressive (BACT) bacterial strains, and an additional 1056 in response to aggressive strain, and 1233 in response to the mild strain (Fig. 2E). Similarly, 2015 genes were differentially regulated in common in 447BACT vs. 450BACT or 447CTL, with an additional 1384 genes showing differential expression in the 447BACT vs. 447CTL treatment (Fig. 2E). Collectively, this suggests that i) the largest transcriptional responses were elicited by the 447BACT treatment, but also that diverse gene sets were stimulated in each of these treatments.

### Functions of top differentially expressed genes

The top 10 up-regulated and down-regulated DEGs for each of the comparisons (Tables 2, 3, 4, 5, 6 and 7, Supp Fig.S4, Supplementary File 1) were used as queries to identify the function of genes. Some of these genes code for proteins with unknown functions, while others were annotated as hypothetical genes, and could be protein coding genes or long non-coding RNA’s (lncRNAs) which are also captured during poly A-tail isolation. In the 450BACT vs. 447BACT comparison, a substantial number of DEGs were identified, with a significant overlap between upregulated genes associated with photosynthesis and defense responses such as LOC542476 (ascorbate peroxidase 2), LOC103651578 (chlorophyll a-b binding protein of LHCII type 1), LOC103644215 (Rubredoxin-like superfamily protein) and LOC100281201 (root phototropism protein two isoform X2). The other top-up-regulated DEGs for 450-BACT vs. 447-BACT include LOC100281213 (pheophorbide an oxygenase), LOC100280107 (PKc_like superfamily protein kinase), and defense response (Zm00001d034461, indole-3-glycerol-phosphate lyase) (Table 2). Furthermore, 3447 downregulated DEGs in 450BACT vs. 447BACT showed significant declines in 450BACT (Table 2). These DGEs were LOC103641099 ((S)-beta-macrocarpene synthase-like), LOC542117 (indole-3-glycerol phosphate lyase, chloroplastic precursor), GRMZM2G064473 (plastid ATP/ADP-transporter), LOC103644033 (cytochrome P450), GRMZM2G150474 (glutathione S-transferase GST 15 isoform X1) and couple of unknown proteins (LOC103650163, LOC103639320 and LOC109943125). These results indicate that in response to infection by the highly aggressive *C. nebraskensis* isolate, the resistant corn line exhibits increased expression of photosynthesis-related and defense-associated genes, while downregulating genes involved in secondary metabolism and stress responses when inoculated with BACT.

The genes coding for thaumatin-like protein (LOC103640644), thaumatin-like protein precursor (LOC100284970), 5-formyltetrahydrofolate cyclo-ligase1 (GRMZM2G001904), lipid-transfer protein 2P (LOC103626125), endochitinase A (LOC100283098), chitinase 1 (LOC100192522), cytochrome P450-like protein (GRMZM5G899349), putative MATE efflux family protein (GRMZM2G079127) and Cytosolic Ascorbate Peroxidase (LOC100283822), were significantly up-regulated in 450BACT compare with 450CTL (Table 3). Additionally, the genes encoding transcriptional regulatory protein algP precursor, phosphoinositide phosphatase SAC2 isoform X2, diacylglycerol kinase one isoform X5 and a couple of unknown proteins were downregulated in 450BACT compared with 450CTL. These results show that the resistant corn line responded to infection by the highly aggressive *C. nebraskensis* isolate by upregulating defense-related genes, including those involved in antifungal activity, oxidative stress response, and lipid transport. Conversely, the downregulation of transcriptional and signaling genes suggests possible regulatory changes that may contribute to the resistance mechanism.

The top DEGs in the comparison between 447BACT vs. 447CTL included (S)-beta-macrocarpene synthase (LOC103641097, LOC103641099 and LOC109943125), thaumatin-like protein, thaumatin-like protein precursor, terpene synthase 6, pathogenesis-related protein PRMS, pathogenesis-related protein ten and cytochrome P450 (Table 4). Interestingly, the putative photosynthesis-related genes showed a significant downregulation in 447BACT compared to 447CTL. These include root phototropism protein two isoform X1, photosynthetic NDH subunit of lumenal location 1, chlorophyll a-b binding protein 2, chlorophyll a-b binding protein of LHCII type 1 (LOC103651577 and LOC103651578) and chlorophyll a-b binding protein 48 (Table 4). These results indicate that the susceptible corn line responds to infection by the highly aggressive *C. nebraskensis* isolate by upregulating defense-related genes, including pathogenesis-related proteins and terpene synthases. However, the significant downregulation of photosynthesis-related genes suggests that infection negatively impacts photosynthetic activity in 447, potentially contributing to its susceptibility.

Moreover, LOC100281838 (chitinase 1), GRMZM5G828581 (integral membrane protein like protein), LOC103639506 (flavin-containing monooxygenase 1), LOC103633426 (protein STRUBBELIG-RECEPTOR FAMILY 6), LOC103641099 ((S)-beta-macrocarpene synthase-like), LOC103633692 (very-long-chain enoyl-CoA reductase), LOC100283098 (endochitinase A), LOC109943125 ((S)-beta-macrocarpene synthase-like) and some uncharacterized protein-coding genes (LOC100191791, LOC100192571, LOC100285997, LOC100501142) were up-regulated in 447BACT and downregulated in 447DOAB. The differential expression patterns observed indicate that genes involved in defense response, membrane integrity, and metabolic processes are specifically upregulated in 447BACT but not in 447DOAB, suggesting a stronger transcriptional response to the highly aggressive isolate.

The top up-regulated genes in 450DOAB compared to 447DOAB encoded for ascorbate peroxidase, rubredoxin-like superfamily protein, ATP synthase delta chain, sigma-like factor 6, WD40 domain, exonuclease-endonuclease-phosphatase (EEP) domain superfamily and transcriptional regulator ICP4unknown protein (Table 5). On the other hand, the putative beta-amylase (LOC100283208 and LOC542472), polyamine oxidase six isoform X2, TRIBOA-glucoside O-methyltransferase BX7, Metallothionein-like protein 2 C and indole-2-monooxygenase coding genes showed downregulation in 450DOAB compared with 447DOAB. These results indicate that the resistant line 450 exhibits increased expression of genes related to oxidative stress response, energy production, and transcriptional regulation in response to DOAB, while the susceptible line 447 shows higher expression of genes involved in carbohydrate metabolism and secondary metabolite production.

The genes coding for (S)-beta-macrocarpene synthase (LOC103641097, LOC103641099), thaumatin-like protein, thaumatin and antifungal thaumatin-like protein, (S)-beta-macrocarpene synthase-like, Pathogenesis-related protein PRMS, CPP synthase, cytochrome P450 (CYP) superfamily pathogenesis-related protein four and EG45-like domain-containing protein were up-regulated in 447DOAB compared with 447CTL. Only three downregulated DEGs were identified in 447DOAB compared with 447CTL. These genes were a Protein Nonresponding to Oxylipins 2, mitochondrial-like isoform two, as well as two unknown genes (GRMZM2G142597 and LOC118472181) (Table 6). The strong upregulation of defense-related genes in 447DOAB compared to 447CTL suggests that even the weakly aggressive isolate triggers a defense response in the susceptible line. However, the limited number of downregulated genes indicates that the overall transcriptional shift is primarily driven by the activation of defense mechanisms rather than suppression of other biological processes.

Only five DEGs were identified for 450DOAB vs. 450CTL. These were genes coding for serine/threonine-protein kinase SAPK4, ZCN7 protein, transparent leaf area peptide, TRZ/ATZ family and CLIP-associated protein. Among these genes, the TRZ/ATZ family and CLIP-associated protein-coding genes were downregulated, and the other three were up-regulated in 450DOAB compared with the control plants (Table 7). This limited gene expression change may indicate that CO450 has a more effective baseline defense mechanism that requires less transcriptional reprogramming upon pathogen challenge.

These results highlight how the resistant line (450) shows minimal transcriptional reprogramming, whereas the susceptible line (447) exhibits strong defense activation even against the mild isolate.

**Table 2:** Log2 (fold change of Fragments Per Kilobase Million value; FPKM value) of top 20 up-regulated differentially expressed genes in 450BACT vs 447BACT

		Up-regulated	
No	Gene ID	Gene Description	Log2FC
1	LOC542476	ascorbate peroxidase 2	7.533467
2	LOC100281201	root phototropism protein 2 isoform X2	7.194733
3	LOC100279975	mixed lineage kinase domain-like protein (MLKL)	6.829467
4	LOC103644215	Rubredoxin-like superfamily protein	6.593167
5	LOC100281213	pheophorbide a oxygenase	6.400433
6	GRMZM2G111411_P01	ran-binding protein 1	6.374167
7	LOC103643102	uncharacterized	6.308833
8	LOC103653580	peptidyl-prolyl cis-trans isomerase-like	6.082733
9	LOC100280107	PKc_like super family (protein kinase)	6.030533
10	LOC542484	sigma-like factor 6	5.968067
11	LOC103651577	chlorophyll a-b binding protein of LHCII type 1	5.908462
12	GRMZM2G124975_P01	WD40 domain	5.880133
13	GRMZM2G324973_P01	ankyrin repeat pseudogene	5.867133
14	LOC103651578	chlorophyll a-b binding protein of LHCII type 1	5.831264
15	GRMZM2G025171_P01	ATP synthase delta chain	5.694267
16	LOC109944045	uncharacterized	5.628421
17	LOC103651575	chlorophyll a-b binding protein of LHCII type 1	5.620233
18	LOC100194272	Serine/arginine-rich splicing factor SR45a	5.311535
19	LOC103650242	LCD1 super family (DNA damage checkpoint protein)	5.308667
20	LOC103642093	40S ribosomal protein S4-like	5.303725
		Down regulated	
No	Gene ID	Gene Description	Log^2^FC
1	LOC103641099	(S)-beta-macrocarpene synthase-like	-8.54184
2	LOC109943125	uncharacterized protein	-7.25453
3	LOC542117	indole-3-glycerol phosphate lyase, chloroplastic precursor	-6.20285
4	GRMZM2G004278_P01	Protein kinase superfamily	-6.13987
5	GRMZM2G064473_P01	plastidic ATP/ADP-transporter	-5.91647
6	LOC103653760	probable LRR receptor-like serine/threonine-protein kinase	-5.84943
7	LOC103644033	cytochrome P450	-5.53133
8	GRMZM2G150474_P01	glutathione S-transferase GST 15 isoform X1	-5.45744
9	LOC103650163	uncharacterized protein	-5.416731
10	LOC103639320	uncharacterized protein	-5.401223

**Table 3:** Log2 (fold change of Fragments Per Kilobase Million value; FPKM value) of top 20 up-regulated differentially expressed genes in 450BACTvs 450CTL

		Up-regulated	
No	Gene ID	Gene Description	Log2FC
1	LOC103640644	thaumatin-like protein	4.980733333
2	LOC100284970	thaumatin-like protein precursor	4.924833333
3	GRMZM2G001904_P01	5-formyltetrahydrofolate cyclo-ligase1	4.143666667
4	GRMZM2G066440_P01	putative DEAD-box ATP-dependent RNA helicase	4.117466667
5	GRMZM2G037209_P04	uncharacterized	3.760533333
6	LOC100282059	metallothionein-like protein 2C	3.506454111
7	LOC103626125	lipid-transfer protein 2P	3.456266667
8	GRMZM5G846548_P01	Protein Root UVB sensitive 3	3.384266667
9	LOC100278544	DUF3527 domain protein	3.344233333
10	LOC100283098	endochitinase A	3.332966667
11	LOC100275439	uncharacterized	3.234252334
12	GRMZM2G027232_P03	50S ribosomal protein	3.187211123
13	LOC100192522	chitinase 1	3.182666667
14	LOC103652813	barwin-like	3.158866667
15	GRMZM5G899349_P02	cytochrome P450-like protein	3.108012354
16	LOC103653770	receptor kinase-like protein Xa21	3.078133333
17	GRMZM2G079127_P01	putative MATE efflux family protein	3.066666667
18	LOC100193626	uncharacterized	3.040566667
19	LOC100283822	APx1 - Cytosolic Ascorbate Peroxidase	3.002855741
20	LOC103649606	lecithin-cholesterol acyltransferase-like 1	2.941233333
		Down regulated	
No	Gene ID	Gene Description	Log2FC
1	GRMZM2G355316_P01	transcriptional regulatory protein algP precursor	-3.57193333
2	LOC100384006	uncharacterized protein	-3.35796666
3	LOC103626515	uncharacterized protein	-3.11276666
4	LOC103639503	phosphoinositide phosphatase SAC2 isoform X2	-3.07862445
5	LOC103644317	protein FAR1-RELATED SEQUENCE 6 isoform X2	-2.87453333
6	LOC109623449	uncharacterized protein	-2.76593333
7	LOC100282719	uncharacterized protein	-2.62243333
8	LOC100272662	uncharacterized protein	-2.56843333
9	LOC103636484	diacylglycerol kinase 1 isoform X5	-2.47266666
10	LOC103646841	uncharacterized protein	-2.35833222

**Table 4:** Log2 (fold change of Fragments Per Kilobase Million value; FPKM value) of top 20 up-regulated differentially expressed genes in 447BACT vs 447CTL

		Up-regulated	
No	Gene ID	Gene Description	Log2FC
1	LOC103641097	(S)-beta-macrocarpene synthase	7.7447555
2	LOC103641099	(S)-beta-macrocarpene synthase-like	7.7327422
3	LOC109943125	(S)-beta-macrocarpene synthase-like	7.7160671
4	LOC103640644	thaumatin-like protein	7.6082332
5	LOC100284970	thaumatin-like protein precursor	7.5992334
6	LOC100276695	osmotin-like protein OSM34	7.5676554
7	LOC103636298	glucan endo-1,3-beta-glucosidase	7.2881672
8	LOC100283098	endochitinase A	7.0806331
9	LOC103638999	5-pentadecatrienyl resorcinol O-methyltransferase	7.0600672
10	AN2	CPP synthase	6.8833331
11	GRMZM2G122654_P01	putative cytochrome P450 superfamily protein	6.7561674
12	LOC542688	terpene synthase 6	6.6980331
13	LOC100272820	pathogenesis-related protein PRMS	6.6968222
14	LOC100281076	Uncharacterized	6.6174331
15	LOC100192522	chitinase 1	6.5699674
16	GRMZM2G075283_P01	pathogenesis-related protein 10	6.5010335
17	LOC103639320	glucan endo-1,3-beta-glucosidase GII-like	6.4269555
18	LOC100277641	Uncharacterized	6.4075334
19	LOC100273457	zealexin A1 synthase	6.3747333
20	LOC100191791	cytochrome P450	6.3687671
		Down regulated	
No	Gene ID	Gene Description	Log2FC
1	LOC100281201	root phototropism protein 2 isoform X1	-4.82394
2	LOC103634474	photosynthetic NDH subunit of lumenal location 1	-3.73183
3	GRMZM2G089812_P01	nuclear transcription factor Y subunit C-1	-3.64957
4	GRMZM2G031143_P07	uncharacterized protein	-2.98855
5	GRMZM2G104549_P01	chlorophyll a-b binding protein 2	-2.93363
6	LOC103651577	chlorophyll a-b binding protein of LHCII type 1	-2.88043
7	LOC103651575	chlorophyll a-b binding protein 48	-2.86627
8	LOC103651578	chlorophyll a-b binding protein of LHCII type 1	-2.85171
9	LOC103649960	uncharacterized protein	-2.80032
10	LOC100191457	uncharacterized protein	-2.59623

**Table 5:** Log2 (fold change of Fragments Per Kilobase Million value; FPKM value) of top 20 up-regulated differentially expressed genes in 450DOAB vs 447DOAB

		Up-regulated	
No	Gene ID	Gene Description	Log2FC
1	LOC542476	ascorbate peroxidase 2	7.522633
2	LOC100281201	root phototropism protein 2 isoform X2	7.451933
3	LOC103644215	rubredoxin-like superfamily protein	6.695633
4	LOC542484	sigma-like factor 6	6.628833
5	GRMZM2G025171_P01	ATP synthase delta chain	6.533811
6	LOC103653580	peptidyl-prolyl cis-trans isomerase-like	6.511520
7	LOC100281213	pheophorbide a oxygenase	6.428111
8	LOC103643102	uncharacterized	6.354433
9	GRMZM2G111411_P01	ran-binding protein 1	6.260067
10	GRMZM2G324973_P01	ankyrin repeat pseudogene	6.213755
11	LOC100280107	uncharacterized	6.026464
12	GRMZM2G124975_P01	WD40 domain	5.981867
13	LOC100279975	mixed lineage kinase domain-like protein (MLKL)	5.825367
14	LOC109944045	uncharacterized	5.779433
15	LOC103642093	40S ribosomal protein S4-like	5.610067
16	GRMZM2G124495_P01	uncharacterized	5.607267
17	LOC100194272	serine/arginine-rich splicing factor SR45a	5.482733
18	LOC103632768	transcriptional regulator ICP4	5.342067
19	LOC103641771	exonuclease-endonuclease-phosphatase (EEP) domain superfamily	5.249265
20	GRMZM2G107380_P01	uncharacterized	5.226344
		Down regulated	
No	Gene ID	Gene Description	Log2FC
1	LOC542117	indole-3-glycerol phosphate lyase, chloroplastic precursor	-9.37603
2	LOC100283208	beta-amylase	-8.22403
3	LOC542472	beta-amylase	-7.34717
4	LOC103632539	polyamine oxidase 6 isoform X2	-7.23132
5	LOC118473498	polyamine oxidase 6-like isoform X2	-7.13573
6	LOC100147731	TRIBOA-glucoside O-methyltransferase BX7	-6.65027
7	LOC100282059	Metallothionein-like protein 2C	-6.58554
8	LOC100192631	indole-2-monooxygenase	-6.35321
9	LOC100277233	uncharacterized	-6.34577
10	LOC103629082	uncharacterized	-6.29527

**Table 6:** Log2 (fold change of Fragments Per Kilobase Million value; FPKM value) of top 20 up-regulated differentially expressed genes in 447DOAB vs 447CTL

		Up-regulated	
No	Gene ID	Gene Description	Log2FC
1	LOC103641097	(S)-beta-macrocarpene synthase	4.650767
2	LOC103640644	thaumatin-like protein	4.640833
3	LOC103641099	(S)-beta-macrocarpene synthase-like	4.640233
4	LOC100284970	thaumatin and antifungal thaumatin-like protein	4.633233
5	LOC109943125	(S)-beta-macrocarpene synthase-like	4.630333
6	LOC100272820	Pathogenesis-related protein PRMS	4.315867
7	LOC103638999	5-pentadecatrienyl resorcinol O-methyltransferase	4.264554
8	LOC100275439	uncharacterized	3.723312
9	LOC542688	terpene synthase 6	3.711567
10	LOC103639320	glucan endo-1,3-beta-glucosidase GII-like	3.574467

**Table 7:** Log2 (fold change of Fragments Per Kilobase Million value; FPKM value) of top 20 up-regulated differentially expressed genes in 450DOAB vs 450CTL

		Up-regulated	
No	Gene ID	Gene Description	Log2FC
1	LOC103646488	serine/threonine-protein kinase SAPK4	1.652754
2	LOC100127518	ZCN7 protein	1.520633
3	LOC542110	transparent leaf area peptide	1.023933
		Down regulated	
No	Gene ID	Gene Description	Log2FC
1	LOC100283572	TRZ/ATZ family	-2.66823
2	LOC103646242	CLIP-associated protein	-1.76433

### Gene ontology (GO) analysis

**Fig. 3:**
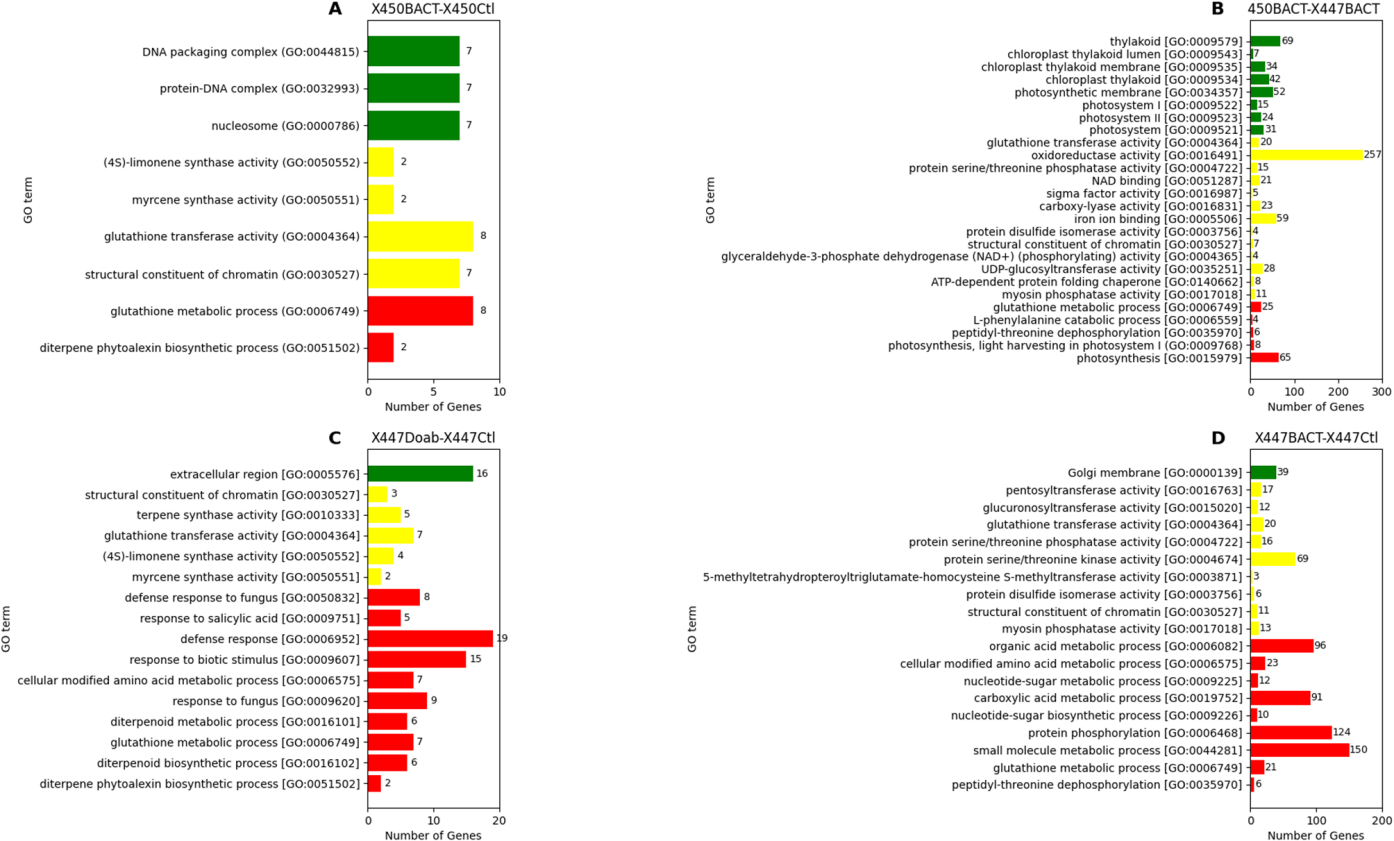
Functional Gene Ontology (GO) categories’ distribution of differentially expressed transcripts. **A**, **B**, **C** and **D** represent GO categories’ distribution of DEGs in 450BACT vs. 450CTL, 450BACT vs. 447BACT, 447DOAB vs. 447CTL, and 447BACT vs. 447CTL, respectively, based on the number of genes in each GO term. Note: Biological process, molecular function, and cellular component categories are presented in red, orange, and green bars

A GO enrichment analysis was conducted using the Maize Genetics and Genomics Database to identify the GO terms associated with the DEGs identified from the six contrasts, assessing the ontology of the gene groups based on their molecular function, biological process, and cellular components. According to our results, most of the DEGs in the comparison of 450BACT vs. 450CTL belonged to the glutathione metabolic process (GO:0006749), as well as the diterpene phytoalexin biosynthetic process (GO:0051502). Meanwhile, in the molecular function category, DEGs were primarily associated with glutathione transferase activity (GO:0004364), structural constituent of chromatin (GO:0030527) and myrcene synthase activity (GO:0050551). In the cellular component category, DEGs are primarily mapped with nucleosome (GO:0000786), protein-DNA complex (GO:0032993) and DNA packaging complex (GO:0044815). The major GO term in molecular function was the protein tyrosine kinase activity (GO:0004713) (Fig. 3A). The enrichment of DEGs in glutathione metabolism and diterpene phytoalexin biosynthesis in 450-BACT vs. 450-CTL suggests that the resistant line (CO450) activates antioxidant and phytoalexin-related defense mechanisms in response to the highly aggressive pathogen. Additionally, the overrepresentation of genes involved in chromatin structure and protein-DNA complexes indicates potential epigenetic regulation of defense responses.

For the comparison between 450BACT vs. 447BACT, our results showed that in the biological process category, the GO terms were mainly associated with photosynthesis (GO:0015979), glutathione metabolic process (GO:0006749), photosynthesis, light harvesting in photosystem I (GO:0009768) and peptidyl-threonine dephosphorylation (GO:0035970). However, in the molecular function category, DEGs were primarily associated with oxidoreductase activity (GO:0016491), iron ion binding (GO:0005506), UDP-glucosyltransferase activity (GO:0035251), carboxy-lyase activity (GO:0016831), NAD binding (GO:0051287) and glutathione transferase activity (GO:0004364). In the cellular component category, DEGs primarily mapped with thylakoid (GO:0009579), photosynthetic membrane (GO:0034357), chloroplast thylakoid (GO:0009534), thylakoid membrane (GO:0042651) and photosystem (GO:0009521) (Fig. 3B). The GO enrichment analysis for 450BACT vs. 447BACT indicates that the resistant corn line 450 activates photosynthesis-related pathways, glutathione metabolism, and antioxidant responses, particularly through oxidoreductase and glutathione transferase activities. Additionally, the strong association with thylakoid and photosystem components suggests an enhanced capacity for photosynthetic processes and light harvesting in response to the pathogen.

Interestingly, in the 447DOAB vs. 447CTL comparison, most of the DEGs were involved in biological processes related to the defense response group, including defense response (GO:0006952), response to biotic stimulus (GO:0009607), response to fungus (GO:0009620) and defense response to fungus (GO:0050832). Our results showed that in 447DOAB vs. 447CTL, the transcripts significantly over-represented among other genes in the molecular process category were glutathione transferase activity (GO:0004364), terpene synthase activity (GO:0010333) and (4 S)-limonene synthase activity (GO:0050552). Interestingly, DEGs were associated only with the extracellular region (GO:0005576) for cellular components (Fig. 3C).

In the comparison of 447BACT vs. 447CTL, the DEGs were associated with biological processes related to small molecule metabolic process (GO:0044281), protein phosphorylation (GO:0006468), organic acid metabolic process (GO:0006082), carboxylic acid metabolic process (GO:0019752) and cellular modified amino acid metabolic process (GO:0006575). In the molecular function category, DEGs were primarily linked to protein serine/threonine kinase activity (GO:0004674), glutathione transferase activity (GO:0004364), protein serine/threonine phosphatase activity (GO:0004722), pentosyltransferase activity (GO:0016763) as well as myosin phosphatase activity (GO:0017018). In the cellular process category, the only transcripts with a high ratio belonged to the Golgi membrane (GO:0000139) (Fig. 3D). The GO enrichment analysis for 447BACT vs. 447CTL revealed a strong association between DEGs and metabolic processes such as small molecule metabolism, protein phosphorylation, and carboxylic acid metabolism.

GO enrichment analysis of DEGs of 450DOAB vs. 447DOAB and 450DOAB vs. 450CTL was null since so few DEGs were identified in these contrasts.

### KEGG enrichment analysis of DEGs

A pathway analysis was carried out to understand better the molecular associations among the DEGs (Tables 8, 9, 10 and 11). The results showed that some DEGs were related to known pathways. In 450BACT vs. 450CTL contrast, nine and eight DEGs were classified into functions related to glutathione metabolism and flavonoid biosynthesis. Based on our results, in 450BACT vs. 447BACT, for up-regulated DEGs, Photosynthesis, Photosynthesis antenna proteins, Carbon fixation in photosynthetic organisms, Porphyrin metabolism, Carotenoid biosynthesis and Nitrogen metabolism were the significantly enriched pathways. On the other hand, for downregulated genes, Glutathione metabolism, Cysteine and methionine metabolism, mitogen-activated protein kinase (MAPK) signaling pathway, Glycolysis/Gluconeogenesis as well as Flavonoid biosynthesis were the significantly enriched pathways. Photosynthesis was the only highly enriched pathway in 450DOAB vs. 447DOAB within the up-regulated genes. However, for downregulated genes, benzoxazinoid (BXD) biosynthesis and glutathione metabolism were the pathways that were significantly enriched. In 447BACT vs. 447CTL, for up-regulated DEGs, 42, 46, 28, 30, 35 and 13 DEGs were classified into MAPK signaling pathway, plant-pathogen interaction, glutathione metabolism, cysteine and methionine metabolism, phenylpropanoid biosynthesis and Flavonoid biosynthesis pathways, respectively. No pathways were assigned to the downregulated DEGs. Our pathway enrichment analysis found no pathways for 450DOAB vs. 450CTL or 447DOAB vs. 447CTL, which had few DEGs overall. The pathway analysis revealed significant molecular associations among DEGs in the different comparisons. Key pathways involved in stress response, such as photosynthesis, glutathione metabolism, and flavonoid biosynthesis, were enriched in several contrasts, particularly in the resistant corn lines. The identification of pathways like MAPK signaling, plant-pathogen interactions, and nitrogen metabolism further emphasizes the potential molecular mechanisms underlying the differential responses of corn lines to bacterial aggressiveness. The absence of significant pathways in the 450DOAB vs. 450CTL and 447DOAB vs. 447CTL contrasts suggest a limited molecular response in these comparisons, which may reflect the smaller number of DEGs detected.

**Table 8:** KEGG enrichment analysis of 450BACT-450CTL

**Treatment**	**Pathway ID**	**Pathway name **	**Number of genes**	***P*** ** value**
Up-regulated				
450BACT-450CTL	zma00941	Flavonoid biosynthesis	8	1.886254e-5
	zma00480	Glutathione metabolism	9	0.003689

**Table 9:** KEGG enrichment analysis of 450BACT-447BACT

**Treatment**	**Pathway ID**	**Pathway name **	**Number of genes**	***P*** ** value**
Up-regulated				
450BACT-447BACT	zma00195	Photosynthesis	48	2.648242e-23
	zma00196	Photosynthesis antenna proteins	13	2.517216e-8
	zma00710	Carbon fixation in photosynthetic organisms	20	4.119686e-5
	zma00860	Porphyrin metabolism	12	0.006541
	zma00906	Carotenoid biosynthesis	9	0.012504
	zma00910	Nitrogen metabolism	9	0.012504
Down regulated				
	zma00480	Glutathione metabolism	27	1.232367e-4
	zma00270	Cysteine and methionine metabolism	28	0.001171
	zma04016	MAPK signaling pathway	33	0.003365
	zma00010	Glycolysis / Gluconeogenesis	29	0.007586
	zma00941	Flavonoid biosynthesis	12	0.035018

**Table 10:** KEGG enrichment analysis of 450DOAB vs 447DOAB

**Treatment**	**Pathway ID**	**Pathway name **	**Number of genes**	***P*** ** value**
Up-regulated				
450 DOAB -447DOAB	zma00195	Photosynthesis	18	0.004505
Down regulated				
	zma00402	Benzoxazinoid biosynthesis	8	1.332787e-6
	zma00480	Glutathione metabolism	16	0.023478

**Table 11:** KEGG enrichment analysis of 447BACT-447CTL

**Treatment**	**Pathway ID**	**Pathway name **	**Number of genes**	***P*** ** value**
Up-regulated				
447BACT-447CTL	zma04016	MAPK signaling pathway	42	5.247055e-7
	zma04626	Plant-pathogen interaction	46	1.830390e-5
	zma00480	Glutathione metabolism	28	6.449941e-5
	zma00270	Cysteine and methionine metabolism	30	2.325305e-4
	zma00940	Phenylpropanoid biosynthesis	35	0.002446
	zma00941	Flavonoid biosynthesis	13	0.010887

### Validation of candidate genes expression

**Fig. 4:**
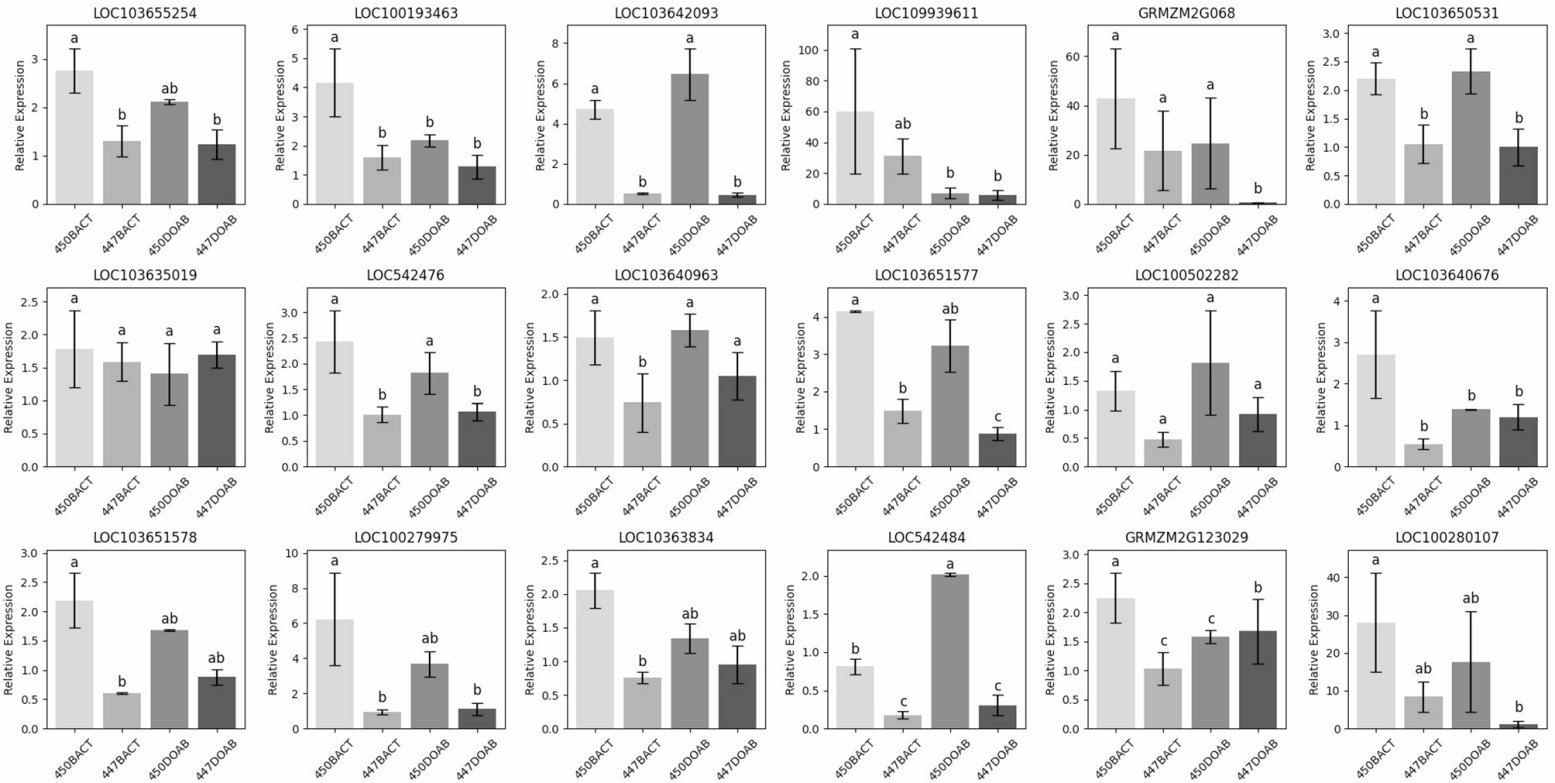
qRT-PCR validation of selected differentially expressed genes. qRT-PCR validation of 18 genes exhibiting differential expression in corn lines 450 and 447 following infection with the highly aggressive (BACT) or weak (DOAB) strains of *Clavibacter nebraskensis*. Actin and beta tubulin was used as a housekeeping gene. The standard error bar shows the standard deviation for three replicate assays

Quantitative Real-Time PCR (qRT-PCR) was used to validate the expression patterns of some of the identified genes. For this purpose, a subset of 18 genes exhibiting high differential gene expression was selected for analysis, and a qRT-PCR performed to assess the expression of the genes in the four treatment groups 447BACT, 447DOAB, 450BACT and 450DOAB relative to the un-inoculated control plants. All loci except one (LOC103635019) exhibited differences in amplification between one or more treatments (Fig. 4), and the qRT-PCR results agreed with the transcriptome (RNA-seq) data for 15 out of 18 genes. These findings further strengthen the reliability of the RNA-seq results and demonstrate the consistency of the identified differential expression patterns across both techniques.

## Discussion

### DEGs and PCA analyses

This study compared gene expression in susceptible and resistant corn lines exposed to highly and weakly aggressive strains of *C. nebraskensis*. The results revealed significant variability in DEGs across treatments, highlighting the dynamic nature of corn-pathogen interactions.

The differences in DEGs between the highly aggressive (BACT) and weakly aggressive (DOAB) bacterial strains in resistant (450) and susceptible (447) corn lines suggest that genetic factors influence the host’s response to varying pathogenic challenges. We observed fewer DEGs in the resistant corn line infected with the weakly aggressive strain, suggesting a more controlled and targeted defense response than the highly aggressive strain. More DEGs were detected in the resistant corn line when challenged by the aggressive strain than in the non-aggressive one (450BACT-447BACT; comparison 4), confirming complex transcriptional reprogramming in response to the highly aggressive pathogen, consistent with the conclusion of Qi et al. [24] that the.

number of DEGs reflects the complexity of the plant’s defense mechanisms.

Down-regulated genes in the susceptible reaction were linked to the salicylic acid (SA) and jasmonic acid (JA) pathways in different ways. For example, increased SA and JA signaling in the resistant reaction suggests their role in the *Rlm1*-mediated resistance. The higher number of DEGs in the resistant corn line indicates a strong defense response, likely mediated through multiple defense pathways. Song et al. [25] infected resistant and susceptible cabbage lines with black rot pathogen and discovered 10,030 DEGs, with 384 overlapping DEGs in both resistant and susceptible lines. They concluded that these overlapping DEGs play essential roles in the early responses of cabbage to the pathogen. Yuan et al. [27] studied host defense mechanisms against maize ear rot caused by *Fusarium graminearum*. They found 487 DEGs in the resistant line and 410 in the susceptible line, indicating a stronger defense response in the resistant line. They also reported that 198 genes were induced in both lines.

Interestingly, infection by a highly aggressive bacterial strain in the resistant corn line upregulated genes related to photosynthesis. This suggests a unique aspect of corn-pathogen interactions, potentially linking photosynthetic processes with the plant’s defense response, as the plant may try to maintain energy production and metabolic functions during pathogenic stress [28]. Previous studies have suggested that photosynthesis-related genes are typically downregulated in response to biotic stress, which promotes the production of reactive oxygen species (ROS) [26, 27]. In contrast, the upregulation of genes involved in JA, SA, and ethylene (ET) synthesis can trigger hormone-mediated pathogen defense response [28]. Specific photosynthesis-related proteins interact with pathogens to regulate defense mechanisms. For example, in *Arabidopsis*, the PSII subunit PsbP binds to the alfalfa mosaic virus protein, inhibiting viral replication [29]. Understanding these interactions could help improve resistance and photosynthetic efficiency in corn cultivars. 

The identification of upregulated defense-related genes, like thaumatin-like proteins and chitinases, highlights the plant’s active defense responses against pathogens [30, 31]. This finding is consistent with the existing literature on plant-pathogen interactions. Singh et al. [32] cloned the AdTLP gene from *Arachis diogoi*, showing its upregulation in response to the late leaf spot pathogen, which enhanced antifungal activity. Transgenic plants expressing AdTLP showed increased resistance to fungal pathogens and improved stress tolerance. Furthermore, another study analyzed leaf transcriptomes of *Malus sieversii* and *M. domestica* after *Valsa mali* attack, identifying five upregulated chitinase genes in *M. sieversii* GH18 genes (MsChi1, MsChi7, MsChi9, MsChi18, and MsChi19), and one in *M. domestica* GH19 gene (MsChi35).

On the contrary, a single *M. sieversii* GH18 gene (MsChi26) was downregulated in response to *V. mali* infection. In *M. domestica*, thirteen genes were upregulated, and four were downregulated in response to the same infection [33].

### GO analysis

#### 450BACT vs. 450CTL

This comparison identified transcripts related to glutathione metabolism and diterpene phytoalexin biosynthesis which underly a molecular response to offset bacterial invasion. Glutathione is an antioxidant critical for maintaining cellular balance during oxidative stress from pathogens [34, 35]. Balotf et al. [36] examined the defense mechanisms in potato roots against *Spongospora subterranea* using transcriptomic and proteomic data. The resistant cultivar revealed increased glutathione metabolism at RNA and protein levels and enhanced lignin metabolism, while the susceptible cultivar upregulated the inositol phosphate pathway. These results imply the importance of glutathione metabolism in disease resistance and provide comprehensive multi-omics data on the *Spongospora*-potato interaction. In this regard, the differential production of diterpenes, as phytoalexins, indicate a targeted defense against bacterial pathogens [37, 38]. The molecular machinery involved in defense is shown by the enrichment of transcripts linked to myrcene synthase activity, the structural component of chromatin, and glutathione transferase activity. Glutathione transferases (GSTs) play a vital role in detoxification processes [39], while chromatin structure modulation might be a regulatory mechanism in the plant’s response to bacterial infection [40]. Myrcene synthase activity suggests the induction of terpenoid biosynthesis, contributing to the plant’s defense mechanisms [41]. Zhou et al. [45] investigated wheat’s intricate Agrobacterium-mediated transformation process, identifying 4,889 DEGs and 90 DEPs. These were primarily linked to metabolism, chromatin assembly/disassembly, and immune defense. Mapping the DEGs revealed dynamic changes in chromatin architecture, suggesting their role in regulating gene expression in response to bacterial challenges [46].

#### 450BACT vs. 447BACT

This comparison identified DEGs linked to photosynthesis, glutathione metabolism, and peptidyl threonine dephosphorylation. This highlights the complex nature of the plant’s response to the highly aggressive pathogens [42]. Activating various molecular functions, including oxidoreductase and glutathione transferase activity, underlines the intricate biochemical pathways involved in the plant’s response to bacterial infection [43, 44].

Liu et al. [48] used RNA sequencing to analyze *Rhizoctonia solani* AG1 IA, identifying 16,453 DEGs with enrichment in carbohydrate metabolism and oxidation-reduction processes. This suggests dynamic changes in cellular structures for photosynthesis as the organism responds to increased resource demands during defense against pathogens. Also, Li et al. [50] found that in wheat lines with varying susceptibility to black point disease caused by *Bipolaris sorokiniana*, those slightly susceptible showed up-regulation of DEGs related to photosynthesis after inoculation.

Key gene ontology enrichment terms highlighted oxidation-reduction, cold response, salt stress, oxidative stress, and cadmium ion mechanisms. Cellular components were primarily linked to the plasma membrane and vesicles, while molecular functions focused on heme binding and peroxidase activity. Antioxidant enzyme activities, including superoxide dismutase, catalase, and peroxidase, were higher in slightly susceptible wheat lines compared to highly susceptible ones. The study suggests that resistance to black point involves managing oxidative stress and energy metabolism, offering new insights into wheat’s resistance mechanisms.

#### 447DOAB vs. 447CTL

The comparison of the susceptible corn line with the weakly aggressive *C. nebraskensis* isolate revealed a strong defense response, evidenced by increased transcript abundance of genes related to defense mechanisms such as reactions to biotic and fungal stimuli. This suggests that the plant prioritizes defense even against weaker threats. Additionally, the prominence of transcripts related to GST activity, terpene synthase, and (4 S)-limonene synthase activity indicates activated enzymatic defenses targeting the bacteria.

Horváth et al. [51] investigated the role of AtGSTF8 and AtGSTU19 in redox-related gene regulation in *Arabidopsis thaliana*. Using wild-type and *Atgst* mutant seedlings treated with various SA concentrations, they found that SA differently affected the expression of redox and oxidative stress genes. *Atgst* mutants showed reduced vitality and altered redox potential, highlighting the role of these GSTs in ROS regulation and redox balance. The study suggests *AtGSTF8* and *AtGSTU19* mediate redox signaling and transcriptional reprogramming in roots.

*OsTPS19*, a terpene synthase gene in rice, enhances resistance to *Magnaporthe oryzae*. Overexpression increases defence, while RNAi generated lines are more susceptible. It produces (S)-limonene, which inhibits spore germination, highlighting its role in pathogen defence [45].

#### 447BACT vs 447CTL

The enrichment of transcripts linked to various metabolic processes and enzyme activities in this comparison suggests extensive metabolic reprogramming during bacterial infection. This reflects the plant’s effort to efficiently allocate resources and regulate defence through kinase/phosphatase signaling and glutathione-mediated responses [46]. The presence of transcripts linked to the Golgi membrane indicates a potential role in membrane-related processes during the defense response against bacterial infection. [47]

### KEGG enrichment analysis of DEGs

#### 450BACT vs. 450CTL

We identified nine and eight DEGs linked to glutathione metabolism and flavonoid biosynthesis respectively indicating a strong activation of these defense pathways during infection of the strong *C. nebraskensis* isolate in the resistant corn line. Yang et al. [48] detected 66, 216, 117, 101, and 27 DEGs in rice corresponding to the top five KEGG-enriched pathways in TH120: phenylpropanoid biosynthesis, plant-pathogen interaction, plant hormone signal transduction, MAPK signaling pathway-plant, and glutathione metabolism, respectively. In addition, Huang et al. [56] examined *Pueraria lobata*’s susceptibility to pseudo-rust disease caused by *Synchytrium puerariae* Miy (SpM). Transcriptomic profiles of two varieties (disease-resistant GUIGE18 and susceptible GUIGE8) were compared after SpM infection. GUIGE18 exhibited more DEGs than GUIGE8, suggesting an earlier and more active response to SpM. A total of 7044 DEGs were identified, with 406 co-expressed DEGs. Transcription factor analysis highlighted the potential roles of the bHLH, WRKY, ERF, and MYB families in the *Pueraria*-pathogen interaction. GO and KEGG enrichment analyses indicated involvement in metabolic, defense response, plant hormone signaling, and flavonoid biosynthesis pathways. In the early response stages, critical gene families (CPK, CESA, PME, CYP) and antioxidase-encoding DEGs were implicated.

#### 450BACT vs. 447BACT

The upregulation of genes involved in photosynthesis, carbon fixation, and related metabolic pathways suggests a complex metabolic reorganization in response to infection of the aggressive strain of bacteria in the resistant vs. susceptible corn lines. The light-harvesting chlorophyll protein complex (LHC) plays a crucial role in regulating the functions of the photosynthetic antenna system [49]. Ye et al., [50] Investigated a resynthesized *Brassica napus* mutant with an inheritable albino phenotype and found that all DEGs encoding LHC were down-regulated in white leaves compared to green leaves, by 3.54- to 21.16-fold. Yellow-green necrosis develops in maize leaves because of chlorophyll’s degradation and ROS accumulation caused by a genetic mutation affecting the coproporphyrinogen III oxidase-encoding gene within the porphyrin pathway [51]. The KEGG pathway associated with porphyrin metabolism was significantly enriched among the DEGs in a peanut blotch-resistant variety 60 h after inoculation [52]. Therefore, this pathway is suggested to enhance resistance to peanut web blotch by triggering a hypersensitive response (HR) mediated by ROS [61].

On the other hand, we observed downregulation of key metabolic and defence pathways in the resistant to susceptible corn lines suggesting that the resistant corn line allocated fewer resources to defense than the susceptible one during infection.

#### 450-DOAB vs. 447-DOAB

The comparison of resistant and susceptible corn lines infected with the weakly aggressive bacterial strain found that the photosynthesis pathway was enriched; the response of the weakly aggressive bacterial strain appears more complex, with no further significant pathway enrichments in the upregulated DEGs. Li et al., [53] Studied the photosynthetic regulatory network in mulberry and detected 6,587 DEGs with 142 genes associated with photosynthesis and chloroplast development. Three genes (*MaCLA1*, *MaTHIC*, and *MaPKP2*) were studied further using the VIGS technique, resulting in altered leaf appearances and reduced expression levels.

On the other hand, in this comparison, downregulation of BXD biosynthesis and glutathione metabolism pathways was observed in response to inoculation of the weak bacterial strains suggesting a modulation of secondary metabolite production and antioxidant defenses. This may reflect a strategic downregulation to conserve resources in the face of a less severe threat. BXDs are prevalent specialized metabolites derived from indole in various monocot crops like wheat, maize, and rye. Their role involves enhancing plant immunity to resist herbivorous arthropods and fungal pathogens [54].

Researchers [55] investigated the stem developmental of the wheat mutant qd and performed GO and KEGG analyses on 5,199 DEGs between qd and wild type. The analyses highlighted biological processes related to protein-DNA complex organization and benzoxazinoid biosynthesis.

#### 447BACT vs. 447Ctl

Inoculation of the susceptible corn line with the aggressive bacterial isolate not surprisingly identified upregulated DEGs in various defence-related pathways, including MAPK signalling, plant-pathogen interaction, glutathione metabolism, cysteine and methionine metabolism, phenylpropanoid biosynthesis, and flavonoid biosynthesis pathways, highlights a comprehensive activation of defence mechanisms during bacterial infection. This suggests a coordinated and robust defense response at both molecular and metabolic levels. The absence of assigned pathways for downregulated DEGs implies a potential focus on upregulating defense mechanisms rather than suppressing specific metabolic pathways during bacterial infection. Ma et al. [56] found that DEGs count increased with continuous peas cropping, so continuous cropping altered the expression of genes involved in plant-pathogen interaction and MAPK signal transduction. It has been reported that the expression of *FLS2* can trigger an immune response in roots [57]. The induction of *FLS2* by *flg22* leads to its binding with *BAK1*, forming a heterologous aggregate. This interaction initiates a signaling cascade involving ROS generation, calcium signaling, MAPK phosphorylation, and gene transcription. Eventually, this cascade produces a defense response [58, 59]. Moreover, it has been stated that treatments of two pea genotypes co-annotate metabolic pathways associated with antioxidant synthesis [56]. This finding follows those of Huang et al. [60] on challenges related to continuous cropping in sugar beet. Jadhav et al. [62] found that potassium silicate treatment significantly enhanced soybean resistance to charcoal rot by upregulating genes involved in key defense pathways, including systemic acquired resistance, head shock protein, MAPK, and detoxification, providing potential targets for genetic improvement and breeding.

## Conclusion

In conclusion, the study comprehensively explored the molecular landscape underlying the interaction between corn and *C. nebraskensis*. The observed variations in gene expression, the interplay of photosynthesis-related genes, and the identification of defense-associated genes contribute valuable knowledge to the field. The robustness of the experimental design, validated through qRT-PCR, enhanced the reliability of the reported findings. The study sets the stage for future investigations, offering insights to inform strategies for developing corn varieties with enhanced resistance to Goss’s bacterial wilt.

### Implications for future research

The findings pave the way for future research to decipher the functional roles of genes with unknown functions. Investigating the specific contributions of these genes to the plant’s defense mechanisms could unravel novel aspects of the molecular dialogue between corn and *C. nebraskensis*. Exploring the functional significance of genes with unknown functions is crucial for advancing our understanding of plant-pathogen interactions. Furthermore, the results of this study have important implications for breeding programs aimed at improving disease resistance in corn. The identification of DEGs associated with defense mechanisms in both susceptible and resistant corn lines provides valuable insights into the molecular pathways underlying resistance to *C. nebraskensis*. These findings can be directly applied to molecular breeding strategies, where genes related to resistance can be used as targets for marker-assisted selection (MAS). For example, genes involved in oxidative stress response, pathogenesis-related proteins, and photosynthesis regulation could be incorporated into breeding programs to develop corn varieties with enhanced resistance to bacterial infections. Furthermore, understanding the differential response of susceptible and resistant lines to both weakly and highly aggressive bacterial strains allow for the development of more resilient varieties that are not only resistant to *C. nebraskensis*, but also potentially to other bacterial pathogens. Ultimately, the knowledge gained from this study could contribute to the development of corn varieties with improved disease resistance, leading to higher yields and more sustainable agricultural practices in regions affected by bacterial pathogens.

## Supplementary Information

Supplementary Material 1: Supplementary File 1, This Excel file contains eight sheets related to the transcriptomic analysis of gene expression in resistant and susceptible corn lines under different treatments with *Clavibacter nebraskensis*. Sheet 1 – Supplementary Table S1: Summary of the number of upregulated and downregulated genes identified in each of the six focal treatment comparisons using DESeq2. DEGs were defined based on an adjusted *p*-value (FDR) < 0.1 and an absolute log2 fold change >1.5. Sheet 2 – FPKM_allsamples: Normalized gene expression data (FPKM values) for all genes across the 18 RNA-seq samples, used as input for downstream analyses such as clustering and visualization. Sheets 3 –8– Full DEG Lists for Six Pairwise Comparisons. Each sheet provides the complete list of differentially expressed genes for one of the six treatment comparisons, including gene IDs, log2 fold changes, and adjusted *p*-values. The six comparisons are as follows: Sheet 3: 447BACT vs 447CTL, Sheet 4: 450BACT vs 450CTL, Sheet 5: 447DOAB vs 447CTL, Sheet 6: 450DOAB vs 450CTL, Sheet 7: 450DOAB vs 447DOAB and sheet 8: 450BACT vs 447BACT.

Supplementary Material 2: Supp Figure S1A, Screen plot depicting the percent variation explained over the first 18 principal components across all 18 RNA-seq samples. S1B, Elbow plot showing the decay within the sum of squares as a function of the number of clusters for an unsupervised clustered heatmap. Figure S1C, hierarchical heatmap of the top 2000 genes exhibiting the highest standard deviation in expression across all samples. Supp Figure S2, Correlation plot showing the 1:1 correlation in gene expression between the mean of all three samples/treatment for all genes between treatments. Supp Figure S3, Volcano plot of the DEGs between control resistant maize (450) and same maize cultivar inoculated with aggressive (BACT) or weak (DOAB) strains of *C. nebraskensis* (450BACT vs 450CTL and 450DOAB vs 450CTL) after five days. Up and down (red) regulated genes that had a log2fold change >1.5 and an FDR *p*-value <0.1 are coloured, while those that are coloured blue had an Adjusted *p*-value<0.1, but a fold change <1.5. Supp Figure S4, Heatmap of top DEGs across all treatments for the corn line 450 (A), corn line 447 (B) or comparing corn lines 447 vs 450 for the weak (DOAB) or aggressive (BACT) bacterial strains (C).

## Data Availability

RNA-seq reads were deposited in NCBI database under project accession PRJNA1204346. All data generated or analyzed during this study are included in this article. The scripts employed to analyze the data are available at: https://github.com/saravictoriagood/Corn_transcriptome.

## Abbreviations

GWLB: Goss’s bacterial wilt and leaf blight
C. nebraskensis: *Clavibacter nebraskensis*
DEGs: Differentially expressed genes
FDR: False discovery rate
PCA: Principal component analysis
