# Supplementary material for: Unveiling molecular mechanisms and candidate genes for goss’s bacterial wilt and leaf blight resistance in corn through RNA-Seq analysis
Source: BMC Genomics. 2025 Aug 18;26:755. doi: 10.1186/s12864-025-11830-4 (PMC12362853; doi:10.1186/s12864-025-11830-4)
Supplement: Supplementary file 2 — Supplementary Material 2: Supp Figure S1A, Screen plot depicting the percent variation explained over the first 18 principal components across all 18 RNA-seq samples. S1B, Elbow plot showing the decay within the sum of squares as a function of the number of clusters for an unsupervised clustered heatmap. Figure S1C, hierarchical heatmap of the top 2000 genes exhibiting the highest standard deviation in expression across all samples. Supp Figure S2, Correlation plot showing the 1:1 correlation in gene expression between the mean of all three samples/treatment for all genes between treatments. Supp Figure S3, Volcano plot of the DEGs between control resistant maize (450) and same maize cultivar inoculated with aggressive (BACT) or weak (DOAB) strains of C. nebraskensis (450BACT vs 450CTL and 450DOAB vs 450CTL) after five days. Up and down (red) regulated genes that had a log2fold change >1.5 and an FDR p-value <0.1 are coloured, while those that are coloured blue had an Adjusted p-value<0.1, but a fold change <1.5. Supp Figure S4, Heatmap of top DEGs across all treatments for the corn line 450 (A), corn line 447 (B) or comparing corn lines 447 vs 450 for the weak (DOAB) or aggressive (BACT) bacterial strains (C). [file 12864_2025_11830_MOESM2_ESM.pdf]

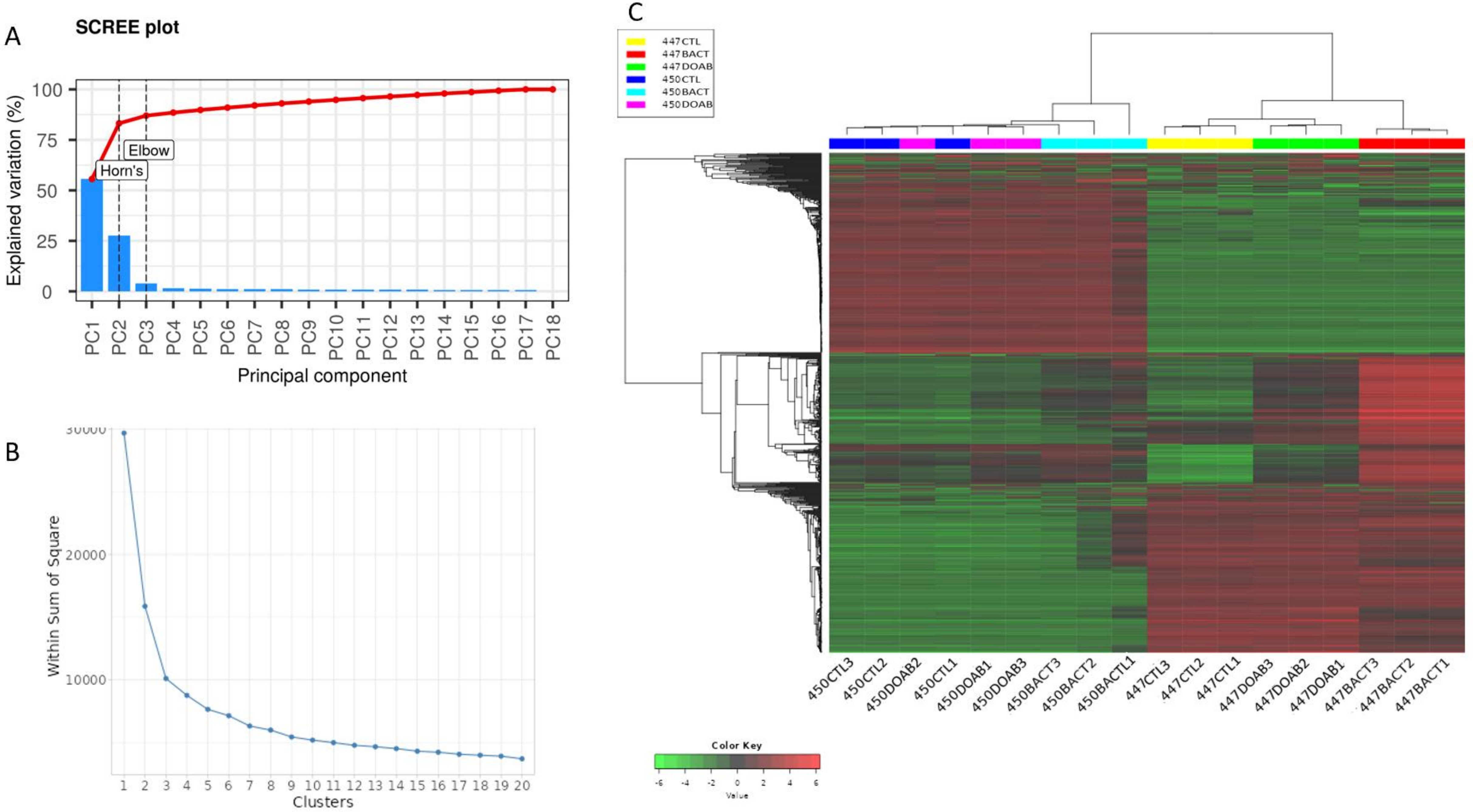

Supp Figure S1

	447CTL1	447CTL2	447CTL3	447BACT1	447BACT2	447BACT3	447DOAB1	447DOAB2	447DOAB3	450CTL1	450CTL2	450CTL3	450BACT1	450BACT2	450BACT3	450DOAB1	450DOAB2	450DOAB3
450DOAB3	0.91	0.91	0.91	0.88	0.88	0.89	0.91	0.91	0.91	0.99	0.99	0.99	0.96	0.98	0.99	0.99	0.99	1
450DOAB2	0.91	0.91	0.91	0.87	0.87	0.88	0.91	0.91	0.91	0.99	0.99	0.99	0.96	0.98	0.99	0.99	1	0.99
450DOAB1	0.91	0.9	0.91	0.88	0.88	0.89	0.91	0.91	0.91	0.98	0.99	0.99	0.96	0.98	0.99	1	0.99	0.99
450BACT3	0.91	0.91	0.91	0.89	0.89	0.9	0.91	0.91	0.91	0.98	0.99	0.99	0.97	0.99	1	0.99	0.99	0.99
450BACT2	0.91	0.91	0.91	0.89	0.9	0.9	0.92	0.92	0.92	0.98	0.98	0.98	0.97	1	0.99	0.98	0.98	0.98
450BACT1	0.93	0.93	0.93	0.9	0.9	0.91	0.93	0.93	0.93	0.96	0.96	0.96	1	0.97	0.97	0.96	0.96	0.96
450CTL3	0.91	0.91	0.91	0.87	0.87	0.88	0.91	0.91	0.91	0.99	0.99	1	0.96	0.98	0.99	0.99	0.99	0.99
450CTL2	0.91	0.9	0.91	0.87	0.87	0.88	0.91	0.91	0.91	0.99	1	0.99	0.96	0.98	0.99	0.99	0.99	0.99
450CTL1	0.91	0.91	0.91	0.86	0.86	0.87	0.91	0.91	0.91	1	0.99	0.99	0.96	0.98	0.98	0.98	0.99	0.99
447DOAB3	0.98	0.98	0.98	0.94	0.94	0.95	0.99	0.99	1	0.91	0.91	0.91	0.93	0.92	0.91	0.91	0.91	0.91
447DOAB2	0.98	0.98	0.98	0.94	0.94	0.95	0.99	1	0.99	0.91	0.91	0.91	0.93	0.92	0.91	0.91	0.91	0.91
447DOAB1	0.98	0.98	0.98	0.95	0.95	0.96	1	0.99	0.99	0.91	0.91	0.91	0.93	0.92	0.91	0.91	0.91	0.91
447BACT3	0.93	0.93	0.93	0.99	0.99	1	0.96	0.95	0.95	0.87	0.88	0.88	0.91	0.9	0.9	0.89	0.88	0.89
447BACT2	0.91	0.92	0.92	0.99	1	0.99	0.95	0.94	0.94	0.86	0.87	0.87	0.9	0.9	0.89	0.88	0.87	0.88
447BACT1	0.91	0.91	0.92	1	0.99	0.99	0.95	0.94	0.94	0.86	0.87	0.87	0.9	0.89	0.89	0.88	0.87	0.88
447CTL3	0.99	0.99	1	0.92	0.92	0.93	0.98	0.98	0.98	0.91	0.91	0.91	0.93	0.91	0.91	0.91	0.91	0.91
447CTL2	0.99	1	0.99	0.91	0.92	0.93	0.98	0.98	0.98	0.91	0.9	0.91	0.93	0.91	0.91	0.9	0.91	0.91
447CTL1	1	0.99	0.99	0.91	0.91	0.93	0.98	0.98	0.98	0.91	0.91	0.91	0.93	0.91	0.91	0.91	0.91	0.91

Supp Figure S2

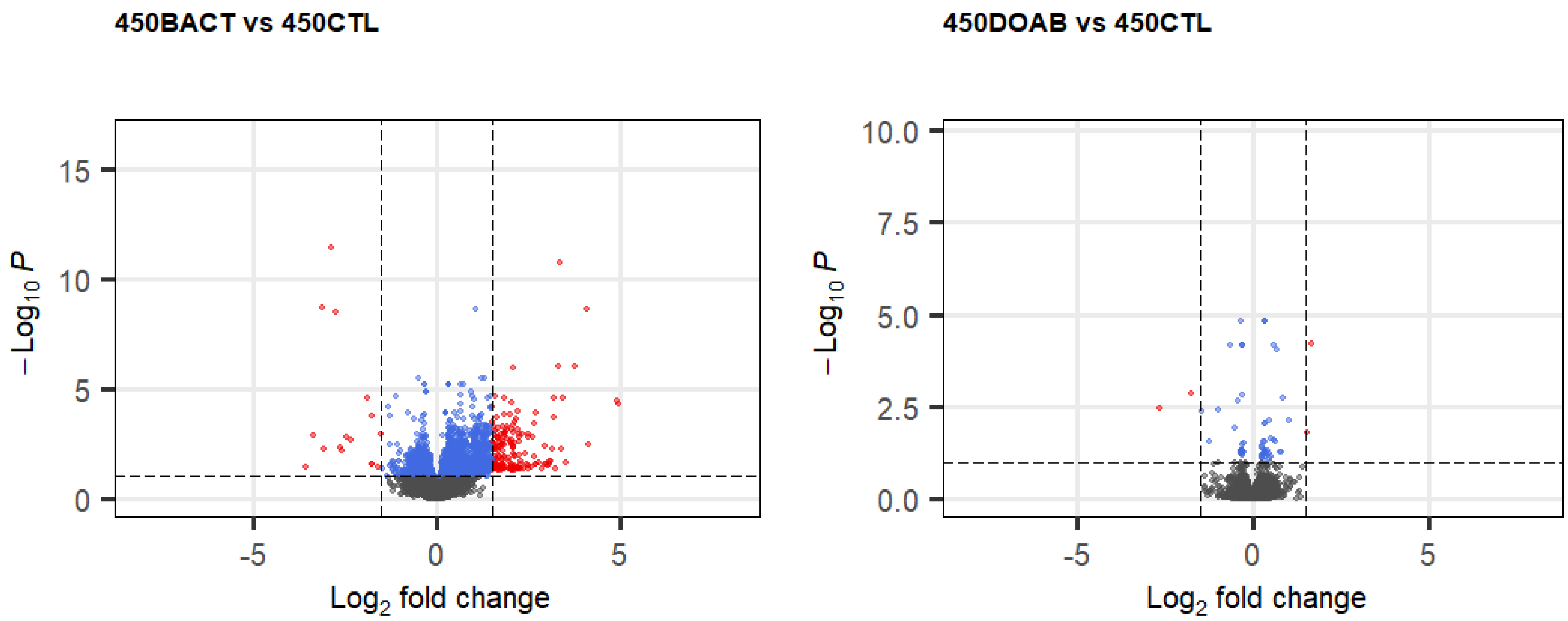

Supp Figure S3

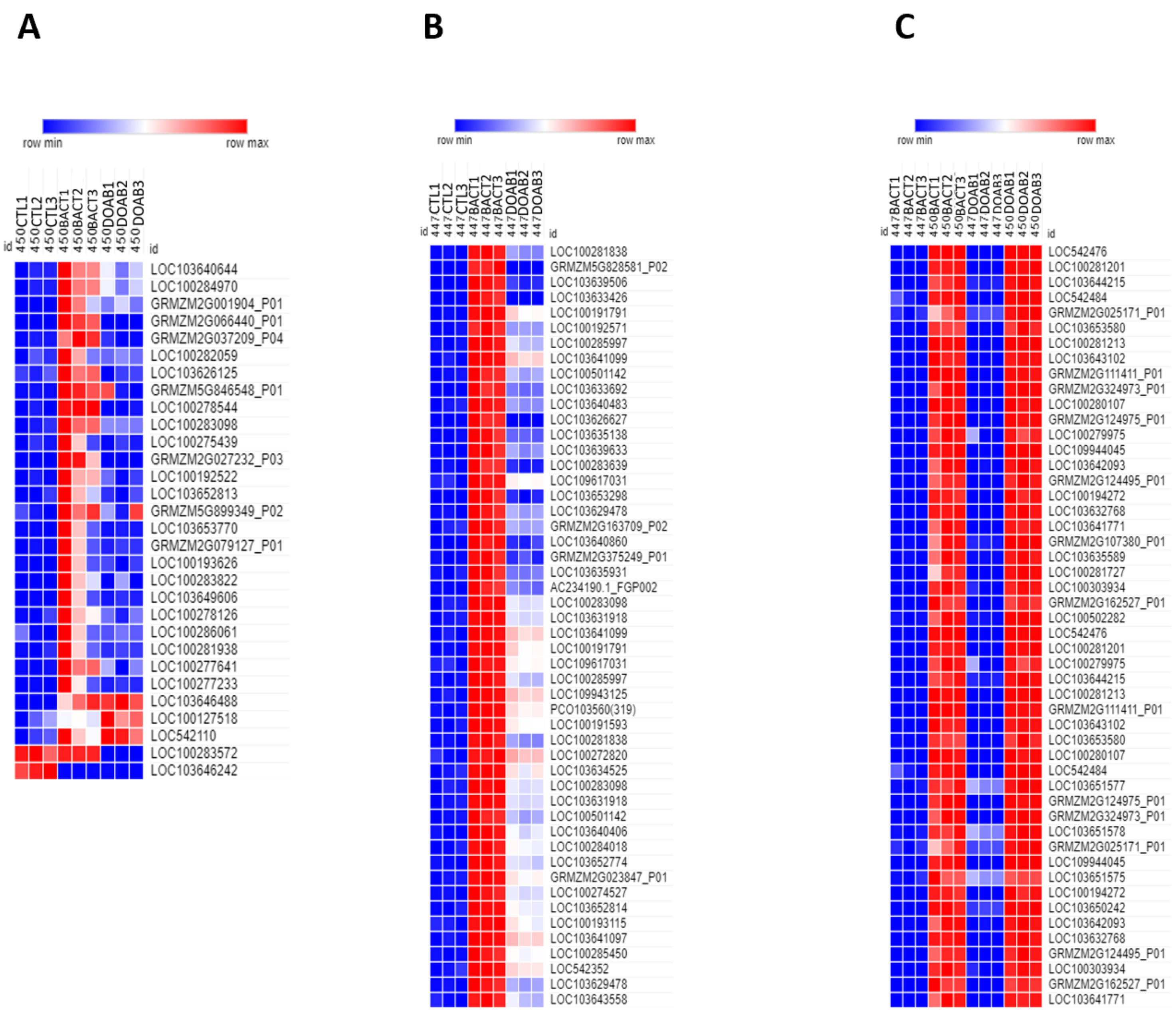

Supp Figure S4
